# Ethyl 2-(3-benzoyl­thio­ureido)acetate

**DOI:** 10.1107/S1600536808024896

**Published:** 2008-08-09

**Authors:** Ibrahim N. Hassan, Bohari M. Yamin, Mohammad B. Kassim

**Affiliations:** a School of Chemical Sciences and Food Technology, Universiti Kebangsaan Malaysia, 43600 Bangi Selangor, Malaysia

## Abstract

The title compound, C_12_H_14_N_2_O_3_S, adopts a *cis*–*trans* geometry of the thio­urea group and is stabilized by intra­molecular hydrogen bonds between the carbonyl O atoms and the H atom of the thio­amide group and by a C—H⋯S interaction. Mol­ecules are linked by two inter­molecular hydrogen bonds (C—H⋯O and N—H⋯O), forming a one-dimensional chain parallel to the *c* axis.

## Related literature

For related literature, see: Allen *et al.* (1987 ▶); Ngah *et al.* (2005 ▶); Yamin & Hassan (2004 ▶); Yamin & Yusof (2003 ▶).
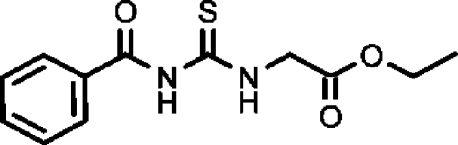


## Experimental

### 

#### Crystal data

C_12_H_14_N_2_O_3_S
*M* *_r_* = 266.31
Monoclinic, 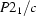

*a* = 11.908 (4) Å
*b* = 7.795 (3) Å
*c* = 14.024 (5) Å
β = 95.600 (5)°
*V* = 1295.5 (8) Å^3^
*Z* = 4
Mo *K*α radiation
μ = 0.25 mm^−1^
*T* = 298 (2) K
0.46 × 0.36 × 0.22 mm

#### Data collection

Bruker SMART APEX CCD area-detector diffractometer
Absorption correction: multi-scan (*SADABS*; Bruker, 2000 ▶) *T*_min_ = 0.893, *T*_max_ = 0.947
6762 measured reflections
2537 independent reflections
1967 reflections with *I* > 2σ(*I*)
*R*_int_ = 0.021

#### Refinement

*R*[*F* ^2^ > 2σ(*F* ^2^)] = 0.041
*wR*(*F* ^2^) = 0.107
*S* = 1.05
2537 reflections
164 parameters
H-atom parameters constrained
Δρ_max_ = 0.20 e Å^−3^
Δρ_min_ = −0.23 e Å^−3^

### 

Data collection: *SMART* (Bruker, 2000 ▶); cell refinement: *SAINT* (Bruker, 2000 ▶); data reduction: *SAINT*; program(s) used to solve structure: *SHELXS97* (Sheldrick, 2008 ▶); program(s) used to refine structure: *SHELXL97* (Sheldrick, 2008 ▶); molecular graphics: *SHELXTL* (Sheldrick, 2008 ▶); software used to prepare material for publication: *SHELXTL*, *PARST* (Nardelli, 1995 ▶) and *PLATON* (Spek, 2003 ▶).

## Supplementary Material

Crystal structure: contains datablocks global, I. DOI: 10.1107/S1600536808024896/at2606sup1.cif

Structure factors: contains datablocks I. DOI: 10.1107/S1600536808024896/at2606Isup2.hkl

Additional supplementary materials:  crystallographic information; 3D view; checkCIF report

## Figures and Tables

**Table 1:** Hydrogen-bond geometry (Å, °)

*D*—H⋯*A*	*D*—H	H⋯*A*	*D*⋯*A*	*D*—H⋯*A*
N2—H2⋯O1	0.86	1.95	2.633 (2)	135
N2—H2⋯O2	0.86	2.43	2.724 (2)	101
C9—H9*B*⋯S1	0.97	2.70	3.045 (2)	101
N1—H1⋯O2^i^	0.86	2.35	3.164 (2)	158
C2—H2*A*⋯O1^i^	0.93	2.51	3.298 (3)	143

Symmetry code: (i) 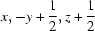.

**Fig. 1.**
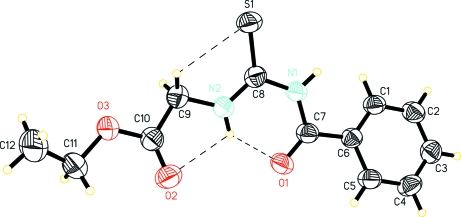
The molecular structure of (I), with displacement ellipsods drawn at the 50% probability level.

**Fig. 2.**
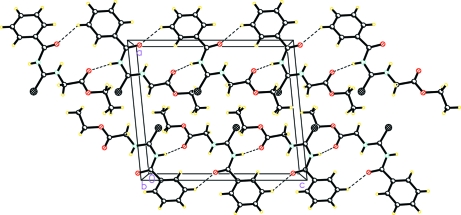
A packing diagram of (I) viewed down the a axis. Hydrogen bonds are shown by dashed lines.

## supplementary crystallographic information

### Comment

Some thiourea derivatives of amino acids, such as 2-[3-(4-methoxbenzoyl) thioureido]-3-methylbutyric acid (Ngah *et al.*, 2005), 3-[3-(4-methoxybenzoyl) thioureido]propanoic acid and (Ngah *et al.*, 2005) and 2-(3-benzoylthioureido) ethanoic dimethyl sulfoxide solvate (II) (Ngah *et al.*, 2005) have been synthesized and their structures have been reported. We are interested to synthesize a series of esters containing thiourea moiety by catalytic transesterification. The title compound, (I), is an ester of (II).

The molecule maintains the *cis*-*trans* geometry of the thiourea moiety (Fig. 1). The phenyl ring (C1—C6) and (S1/N1/N2/O1/C6/C7/C8/C9) fragments are each planar with maximum deviation of 0.031 (2)Å for C6 atom from the least square plane of the later. The dihedral angle between the two planes is 26.53 (8)°. The bond lengths and angles are in normal ranges (Allen *et al.*, 1987). There are three intramolecular hydrogen bonds, N2—H2···O1, N2—H2···O2 and C9—H9B···S1. As a result, one pseudo-six-membered ring (N2/H2/O1/C7/N1/C8) and two pseudo-five-member ring (N2/H2/O2/C10/C9) and (C9/H9B/S1/C8/N2) are formed, respectively. In the crystal structure, the molecules are linked by N1—H1···O2 and C9—H9B···S1 intermolecular hydrogen bonds to form one dimensional chain along the *c* axis (Fig. 2).

### Experimental

2-(3-Benzoylthioureido)ethanoic acid was prepared as reported (Ngah *et al.*, 2005). 2.38 g (10 mmol) of 2-(3-benzoylthioureido)ethanoic acid and 2.45 g (10 mmol) of lanthanum chloride were refluxed in methanol for 17 h. The resulting solution was left for one day at room temperature. Recrystallization of the resulting solid from dichloromethane gave colourless crystals of (I) [yield: 70%]).

### Refinement

All H-atoms attached to C were positioned geometrically and refined using a riding model *U*_iso_=1.2*U*_eq_ (C) for aromatic 0.93 Å, *U*_iso_ = 1.2*U*_eq_ (C) for CH_2_ 0.97 Å and *U*_iso_ = 1.5*U*_eq_ (C) for CH_3_ 0.97 Å. Hydrogen atoms attached to N were also positioned geometrically and allowed to ride on their parent atoms and with *U*_iso_(H) = 1.2*U*_eq_(N) for N–H 0.86 Å.

### Crystal data

**Table:**

C_12_H_14_N_2_O_3_S	*F*_000_ = 560
*M**_r_* = 266.31	*D*_x_ = 1.365 Mg m^−^^3^
Monoclinic, *P*2_1_/*c*	Mo *K*α radiation λ = 0.71073 Å
Hall symbol: -P 2ybc	Cell parameters from 2409 reflections
*a* = 11.908 (4) Å	θ = 1.7–26.0º
*b* = 7.795 (3) Å	µ = 0.25 mm^−^^1^
*c* = 14.024 (5) Å	*T* = 298 (2) K
β = 95.600 (5)º	Block, colourless
*V* = 1295.5 (8) Å^3^	0.46 × 0.36 × 0.22 mm
*Z* = 4	

### Data collection

**Table:**

Bruker SMART APEX CCD area-detector diffractometer	2537 independent reflections
Radiation source: fine-focus sealed tube	1967 reflections with *I* > 2σ(*I*)
Monochromator: graphite	*R*_int_ = 0.021
*T* = 298(2) K	θ_max_ = 26.0º
ω scans	θ_min_ = 1.7º
Absorption correction: multi-scan(SADABS; Bruker, 2000)	*h* = −14→13
*T*_min_ = 0.893, *T*_max_ = 0.947	*k* = −9→9
6762 measured reflections	*l* = −16→17

### Refinement

**Table:**

Refinement on *F*^2^	Secondary atom site location: difference Fourier map
Least-squares matrix: full	Hydrogen site location: inferred from neighbouring sites
*R*[*F*^2^ > 2σ(*F*^2^)] = 0.041	H-atom parameters constrained
*wR*(*F*^2^) = 0.107	*w* = 1/[σ^2^(*F*_o_^2^) + (0.0476*P*)^2^ + 0.3616*P*] where *P* = (*F*_o_^2^ + 2*F*_c_^2^)/3
*S* = 1.06	(Δ/σ)_max_ < 0.001
2537 reflections	Δρ_max_ = 0.20 e Å^−^^3^
164 parameters	Δρ_min_ = −0.23 e Å^−^^3^
Primary atom site location: structure-invariant direct methods	Extinction correction: none

### Special details

**Table:**

Geometry. All e.s.d.'s (except the e.s.d. in the dihedral angle between two l.s. planes) are estimated using the full covariance matrix. The cell e.s.d.'s are taken into account individually in the estimation of e.s.d.'s in distances, angles and torsion angles; correlations between e.s.d.'s in cell parameters are only used when they are defined by crystal symmetry. An approximate (isotropic) treatment of cell e.s.d.'s is used for estimating e.s.d.'s involving l.s. planes.
Refinement. Refinement of *F*^2^ against ALL reflections. The weighted *R*-factor *wR* and goodness of fit *S* are based on *F*^2^, conventional *R*-factors *R* are based on *F*, with *F* set to zero for negative *F*^2^. The threshold expression of *F*^2^ > σ(*F*^2^) is used only for calculating *R*-factors(gt) *etc*. and is not relevant to the choice of reflections for refinement. *R*-factors based on *F*^2^ are statistically about twice as large as those based on *F*, and *R*- factors based on ALL data will be even larger.

### Fractional atomic coordinates and isotropic or equivalent isotropic displacement parameters (Å^2^)

**Table:**

	*x*	*y*	*z*	*U*_iso_*/*U*_eq_	
S1	0.36516 (4)	0.11910 (9)	0.60927 (4)	0.0632 (2)	
O1	0.01958 (11)	0.1765 (2)	0.44591 (9)	0.0615 (4)	
O2	0.21706 (12)	0.0629 (2)	0.25554 (10)	0.0657 (4)	
O3	0.37578 (11)	−0.0928 (2)	0.25994 (9)	0.0626 (4)	
N1	0.15088 (12)	0.2028 (2)	0.57452 (10)	0.0454 (4)	
H1	0.1613	0.2428	0.6319	0.055*	
N2	0.22969 (13)	0.0690 (2)	0.45050 (11)	0.0506 (4)	
H2	0.1650	0.0821	0.4183	0.061*	
C1	−0.03537 (15)	0.2974 (3)	0.68490 (13)	0.0482 (5)	
H1A	0.0242	0.2412	0.7196	0.058*	
C2	−0.11815 (16)	0.3747 (3)	0.73213 (14)	0.0540 (5)	
H2A	−0.1145	0.3696	0.7986	0.065*	
C3	−0.20575 (16)	0.4590 (3)	0.68161 (15)	0.0545 (5)	
H3	−0.2610	0.5118	0.7139	0.065*	
C4	−0.21224 (16)	0.4658 (3)	0.58254 (15)	0.0564 (5)	
H4	−0.2714	0.5237	0.5483	0.068*	
C5	−0.13077 (15)	0.3864 (3)	0.53472 (14)	0.0506 (5)	
H5	−0.1361	0.3887	0.4681	0.061*	
C6	−0.04087 (14)	0.3032 (2)	0.58545 (12)	0.0417 (4)	
C7	0.04366 (15)	0.2218 (3)	0.52873 (13)	0.0455 (4)	
C8	0.24393 (15)	0.1270 (2)	0.53931 (13)	0.0438 (4)	
C9	0.31818 (16)	−0.0154 (3)	0.40544 (13)	0.0530 (5)	
H9A	0.3234	−0.1342	0.4260	0.064*	
H9B	0.3898	0.0398	0.4250	0.064*	
C10	0.29539 (15)	−0.0079 (3)	0.29841 (13)	0.0477 (5)	
C11	0.36926 (19)	−0.1060 (4)	0.15628 (15)	0.0689 (6)	
H11A	0.3349	−0.0035	0.1270	0.083*	
H11B	0.3236	−0.2041	0.1345	0.083*	
C12	0.4835 (2)	−0.1258 (5)	0.1294 (2)	0.1037 (11)	
H12A	0.5247	−0.0212	0.1424	0.155*	
H12B	0.4808	−0.1518	0.0623	0.155*	
H12C	0.5205	−0.2176	0.1658	0.155*	

### Atomic displacement parameters (Å^2^)

**Table:**

	*U*^11^	*U*^22^	*U*^33^	*U*^12^	*U*^13^	*U*^23^
S1	0.0420 (3)	0.0982 (5)	0.0482 (3)	0.0141 (3)	−0.0011 (2)	−0.0037 (3)
O1	0.0460 (8)	0.0913 (11)	0.0454 (8)	0.0093 (7)	−0.0038 (6)	−0.0176 (7)
O2	0.0519 (8)	0.0904 (12)	0.0549 (8)	0.0172 (8)	0.0059 (7)	0.0103 (8)
O3	0.0517 (8)	0.0912 (11)	0.0451 (8)	0.0174 (8)	0.0064 (6)	−0.0090 (7)
N1	0.0373 (8)	0.0613 (11)	0.0374 (8)	0.0036 (7)	0.0020 (6)	−0.0044 (7)
N2	0.0394 (8)	0.0679 (11)	0.0440 (9)	0.0061 (8)	0.0027 (7)	−0.0091 (8)
C1	0.0392 (9)	0.0605 (13)	0.0441 (10)	0.0027 (9)	−0.0004 (8)	0.0007 (9)
C2	0.0479 (11)	0.0718 (14)	0.0423 (10)	−0.0006 (10)	0.0049 (8)	−0.0038 (10)
C3	0.0431 (10)	0.0649 (14)	0.0567 (12)	0.0026 (10)	0.0109 (9)	−0.0082 (10)
C4	0.0413 (10)	0.0695 (14)	0.0582 (12)	0.0115 (10)	0.0030 (9)	0.0066 (10)
C5	0.0427 (10)	0.0663 (14)	0.0420 (10)	0.0027 (10)	0.0005 (8)	0.0031 (9)
C6	0.0340 (9)	0.0472 (11)	0.0437 (9)	−0.0023 (8)	0.0020 (7)	−0.0009 (8)
C7	0.0403 (9)	0.0530 (12)	0.0425 (10)	−0.0013 (8)	0.0000 (8)	−0.0003 (8)
C8	0.0403 (9)	0.0491 (11)	0.0426 (10)	0.0020 (8)	0.0069 (8)	0.0048 (8)
C9	0.0486 (11)	0.0632 (14)	0.0479 (11)	0.0103 (10)	0.0078 (9)	−0.0017 (10)
C10	0.0407 (10)	0.0548 (12)	0.0480 (10)	−0.0036 (9)	0.0075 (8)	−0.0022 (9)
C11	0.0648 (13)	0.0956 (18)	0.0464 (12)	0.0088 (13)	0.0057 (10)	−0.0100 (12)
C12	0.0712 (17)	0.176 (3)	0.0673 (16)	0.0198 (19)	0.0224 (13)	−0.0087 (19)

### Geometric parameters (Å, °)

**Table:**

S1—C8	1.6656 (19)	C3—C4	1.385 (3)
O1—C7	1.221 (2)	C3—H3	0.9300
O2—C10	1.194 (2)	C4—C5	1.379 (3)
O3—C10	1.322 (2)	C4—H4	0.9300
O3—C11	1.452 (2)	C5—C6	1.387 (3)
N1—C7	1.380 (2)	C5—H5	0.9300
N1—C8	1.388 (2)	C6—C7	1.485 (3)
N1—H1	0.8600	C9—C10	1.500 (3)
N2—C8	1.320 (2)	C9—H9A	0.9700
N2—C9	1.439 (2)	C9—H9B	0.9700
N2—H2	0.8600	C11—C12	1.455 (3)
C1—C2	1.379 (3)	C11—H11A	0.9700
C1—C6	1.390 (2)	C11—H11B	0.9700
C1—H1A	0.9300	C12—H12A	0.9600
C2—C3	1.370 (3)	C12—H12B	0.9600
C2—H2A	0.9300	C12—H12C	0.9600
			
C10—O3—C11	118.29 (16)	O1—C7—C6	121.65 (16)
C7—N1—C8	127.91 (15)	N1—C7—C6	116.19 (15)
C7—N1—H1	116.0	N2—C8—N1	116.61 (15)
C8—N1—H1	116.0	N2—C8—S1	124.54 (14)
C8—N2—C9	122.58 (15)	N1—C8—S1	118.83 (14)
C8—N2—H2	118.7	N2—C9—C10	110.66 (16)
C9—N2—H2	118.7	N2—C9—H9A	109.5
C2—C1—C6	120.11 (17)	C10—C9—H9A	109.5
C2—C1—H1A	119.9	N2—C9—H9B	109.5
C6—C1—H1A	119.9	C10—C9—H9B	109.5
C3—C2—C1	120.33 (18)	H9A—C9—H9B	108.1
C3—C2—H2A	119.8	O2—C10—O3	125.96 (18)
C1—C2—H2A	119.8	O2—C10—C9	125.28 (18)
C2—C3—C4	120.14 (18)	O3—C10—C9	108.75 (16)
C2—C3—H3	119.9	O3—C11—C12	107.9 (2)
C4—C3—H3	119.9	O3—C11—H11A	110.1
C5—C4—C3	119.86 (18)	C12—C11—H11A	110.1
C5—C4—H4	120.1	O3—C11—H11B	110.1
C3—C4—H4	120.1	C12—C11—H11B	110.1
C4—C5—C6	120.32 (18)	H11A—C11—H11B	108.4
C4—C5—H5	119.8	C11—C12—H12A	109.5
C6—C5—H5	119.8	C11—C12—H12B	109.5
C5—C6—C1	119.22 (17)	H12A—C12—H12B	109.5
C5—C6—C7	117.04 (16)	C11—C12—H12C	109.5
C1—C6—C7	123.72 (16)	H12A—C12—H12C	109.5
O1—C7—N1	122.16 (17)	H12B—C12—H12C	109.5
			
C6—C1—C2—C3	−0.6 (3)	C5—C6—C7—N1	154.41 (18)
C1—C2—C3—C4	0.6 (3)	C1—C6—C7—N1	−26.8 (3)
C2—C3—C4—C5	0.5 (3)	C9—N2—C8—N1	−178.79 (17)
C3—C4—C5—C6	−1.5 (3)	C9—N2—C8—S1	2.7 (3)
C4—C5—C6—C1	1.5 (3)	C7—N1—C8—N2	1.6 (3)
C4—C5—C6—C7	−179.66 (19)	C7—N1—C8—S1	−179.77 (16)
C2—C1—C6—C5	−0.5 (3)	C8—N2—C9—C10	−158.69 (18)
C2—C1—C6—C7	−179.21 (19)	C11—O3—C10—O2	−1.2 (3)
C8—N1—C7—O1	−3.0 (3)	C11—O3—C10—C9	178.55 (19)
C8—N1—C7—C6	177.93 (17)	N2—C9—C10—O2	2.4 (3)
C5—C6—C7—O1	−24.7 (3)	N2—C9—C10—O3	−177.34 (17)
C1—C6—C7—O1	154.1 (2)	C10—O3—C11—C12	153.0 (2)

### Hydrogen-bond geometry (Å, °)

**Table:**

*D*—H···*A*	*D*—H	H···*A*	*D*···*A*	*D*—H···*A*
N2—H2···O1	0.86	1.95	2.633 (2)	135
N2—H2···O2	0.86	2.43	2.724 (2)	101
C9—H9B···S1	0.97	2.70	3.045 (2)	101
N1—H1···O2^i^	0.86	2.35	3.164 (2)	158
C2—H2A···O1^i^	0.93	2.51	3.298 (3)	143

Symmetry codes:  (i) *x*, −*y*+1/2, *z*+1/2.
